# Galantamine protects against lipopolysaccharide-induced acute lung injury in rats

**DOI:** 10.1590/1414-431X20155008

**Published:** 2015-12-04

**Authors:** G. Li, CL. Zhou, QS. Zhou, HD. Zou

**Affiliations:** Department of Critical Care Medicine, Renmin Hospital, Wuhan University, Wuhan, Hubei Province, China

**Keywords:** Galantamine, Acute lung injury, Lipopolysaccharide, HMGB1

## Abstract

Lipopolysaccharide (LPS)-induced endotoxemia triggers the secretion of proinflammatory cytokines and can cause acute lung injury (ALI). The high mobility group box 1 (HMGB1) protein plays an important role as a late mediator of sepsis and ALI. Galantamine (GAL) is a central acetylcholinesterase inhibitor that inhibits the expression of HMGB1. This study evaluated the effects of GAL by measuring levels of inflammatory mediators and observing histopathological features associated with LPS-induced ALI. Sixty 8-10 week old male Sprague-Dawley rats (200-240 g) were randomized into three groups as follows: control group, LPS group (7.5 mg/kg LPS), and LPS+GAL group (5 mg/kg GAL before LPS administration). Histopathological examination of lung specimens obtained 12 h after LPS administration was performed to analyze changes in wet-to-dry (W/D) weight ratio, myeloperoxidase (MPO) activity, and HMGB1 expression level. Additionally, plasma concentrations of tumor necrosis factor-α, interleukin-6, and HMGB1 were measured using an enzyme-linked immunosorbent assay at 0 (baseline), 3, 6, 9, and 12 h after LPS administration. Mortality in the three groups was recorded at 72 h. LPS-induced ALI was characterized by distortion of pulmonary architecture and elevation of MPO activity, W/D weight ratio, and levels of pro-inflammatory cytokines, including tumor necrosis factor-α, interleukin-6, and HMGB1. Pretreatment with GAL significantly reduced the LPS-induced lung pathological changes, W/D weight ratio, levels of pro-inflammatory cytokines and MPO activity (ANOVA). Moreover, GAL treatment significantly decreased the mortality rate (ANOVA). In conclusion, we demonstrated that GAL exerted a protective effect on LPS-induced ALI in rats.

## Introduction

Acute lung injury (ALI) is a leading cause of death in patients with sepsis, and has shown an annual increase in incidence over the past few years (1,2). Despite remarkable advances in sepsis treatment, the occurrence of ALI and subsequent acute respiratory failure in critically ill patients remains unacceptably high (3).

The exact nature of the cell signaling pathways involved in the pathophysiology of sepsis-induced ALI remains elusive. However, cumulative evidence has suggested that the process is mediated by increased pulmonary expression of pro-inflammatory cytokines, such as tumor necrosis factor (TNF)-α, interleukin (IL)-1, IL-6, and high mobility group box 1 (HMGB1) (4). These pro-inflammatory cytokines are believed to trigger, amplify, and perpetuate the inflammatory response, thereby affecting gas exchange and causing refractory hypoxemia.

HMGB1 is an intranuclear protein that was initially recognized to be crucial for the regulation of gene transcription and stabilization of the nucleosome. Extracellular HMGB1 released from necrotic tissue and activated monocytes and macrophages functions as a late mediator in ALI secondary to sepsis. HMGB1 is reported to be involved in neutrophil accumulation, interstitial edema, disruption of epithelial integrity, leakage of proteins into the alveolar space, and increased production of pro-inflammatory cytokines associated with the pathogenesis of ALI (5,6). In addition, anti-HMGB1 antibodies prevented death in experimental mice with sepsis and the resultant ALI (7,8), indicating that therapeutic agents that attenuate HMGB1 release may have potential for the prevention and treatment of ALI.

Galantamine (GAL) is a competitive and reversible cholinesterase inhibitor that is used in the management of Alzheimer's disease and other conditions involving memory impairment (9,10). Recent studies showed that GAL attenuated the severity of local inflammation in animals, suppressed the degree of systemic inflammatory response elicited by lipopolysaccharide (LPS), and inhibited TNF-α expression in rats with LPS-induced peritonitis (11,12). The anti-inflammatory action of GAL might be mediated by the cholinergic nervous system, which exerts its effects via the vagus nerve and functions as a natural anti-inflammatory system to prevent the excessive release of inflammatory cytokines in the event of infection, sepsis, or autoimmune diseases such as rheumatoid arthritis (13,14). Recent studies have shown that the nicotinic acetylcholine receptor alpha7 subunit plays an important role in the cholinergic anti-inflammatory effect (15). GAL, which is extracted from the bulb of the snowdrop flower, is an agonist to nicotinic acetylcholine receptors, and therefore is expected to enhance the cholinergic anti-inflammatory pathway. However, it remains to be determined whether GAL can exert anti-inflammatory effects to reduce the severity of ALI secondary to sepsis.

The present study investigated whether GAL inhibits the production of inflammatory cytokines and reduces the severity of LPS-induced ALI.

## Material and Methods

### Ethics statement

All of the experimental procedures performed in this study were in accordance with the Guide for the Care and Use of Laboratory Animals, proposed by the National Institutes of Health. The study protocol was approved by the animal experimental Ethics Committee of Wuhan University, China.

### Experiment animals

A total of 90 8-10 week old male Sprague-Dawley rats (200-240 g) were used in this study. All experimental animals were obtained from the Experiment Animal Center, Wuhan University (permit number: Hubei 00001306). The rats were housed in cages maintained at room temperature with a 12-h light/dark cycle. They were fed with standard pellet diet and tap water *ad libitum*.

### Reagents

*Escherichia coli* 0111:B4 endotoxin was purchased from Sigma-Aldrich (USA). GAL was purchased from EMD Biosciences Inc. (USA). The rabbit anti-HMGB1 polyclonal antibody was obtained from Boster Biotechnology Co. (China), and antibodies for Western blotting were purchased from Pierce (Pierce Biotechnology, USA). The kit to determine HMGB1 expression using the streptavidin-biotic complex method was obtained from Boster Biotechnology Co. The myeloperoxidase (MPO) activity kit was obtained from Jiancheng Bioengineering Institute (China) and the cytokine immunoassay kits were purchased from R&D Systems (USA).

### Experimental protocols

Rats were randomized into three groups: LPS group (n=30), in which LPS (7.5 mg/kg, dissolved in 0.5 mL sterile saline) was administered by an intravenous (iv) injection via the tail vein; LPS+GAL group (n=30), in which GAL (5 mg/kg, intraperitoneal, ip) was administered 30 min before injection of LPS (7.5 mg/kg, dissolved in 0.5 mL sterile saline, iv); and a control group (n=30), in which the rats were administered saline at a volume equivalent to that in the other groups. Ten rats in each group were separately investigated as a subgroup for survival analysis. Rats were euthanized with an overdose of sodium pentobarbital (100 mg/kg, ip). Then, lung tissue specimens and blood samples were obtained for further analysis.

### Survival study

To determine the mortality rate, the survival rate in all three groups was assessed at 72 h after the administration of LPS.

### Histologic analysis

Twelve hours after LPS administration, the rats were euthanized (n=5, 3, and 5 in the control, LPS, and LPS+GAL groups, respectively). The obtained lung tissue specimens were fixed with 10% formalin, embedded in paraffin, cut into 5 μm-thick sections and mounted onto slides. The sections were then stained with hematoxylin and eosin (H&E) as per the standard staining method (16). Histologic changes were graded by a pathologist blinded to the clinical status of the rats. The lung tissue samples were then scored for the degree of intra-alveolar edema, intra-alveolar hemorrhage, and neutrophil infiltration using grades 0 to 4 (0, absent; 1, mild; 2, moderate; 3, severe; 4, overwhelming) with a maximum score of 12, as described previously (17).

### Wet-to-dry weight ratio

After the animals were euthanized at 12 h, the chest cavity was opened and the right lung was ligated and excised. The lung specimen was then rinsed briefly in phosphate buffered saline (PBS), blotted, and weighed to determine the ‘wet’ weight. Subsequently, the lungs were dried in an oven at 80°C for 48 h to obtain the ‘dry’ weight. The ratio of wet-to-dry (W/D) weight was then calculated.

### MPO assay

The level of MPO activity in the lung parenchyma, which is a marker of the extent of neutrophil infiltration, was measured 12 h after LPS administration, by using a modified version of a previously described method (18). In brief, frozen lung specimens were weighed and homogenized in hexadecyltrimethylammonium bromide (HTAB) buffer (0.5% HTAB in 50 mM phosphate buffer, pH 6.0). The supernatant of the homogenate was then collected after sonication and centrifugation at 40,000 *g* for 15 min. MPO activity was determined by measuring the H_2_O_2_-dependent oxidation of *ο*-dianisidinehydrochloride in a 96-well plate reader, at 460 nm. MPO activity was expressed per gram of lung weight.

### Immunohistochemical analysis for HMGB1

The lung sections were immunostained for HMGB1 using the streptavidin-biotin complex. All steps, including deparaffinization and counterstaining with hematoxylin, were performed in accordance with the manufacturer's instructions. Non-specific binding of the antibodies was blocked by incubating them with 100 µL of 5% normal goat serum for 20 min. Then, the lung sections were incubated overnight at 4°C in the presence of mouse anti-rat HMGB1 polyclonal antibody (1:1000) as the primary antibody, washed with phosphate buffered saline, and incubated in the presence of anti-mouse IgG antibody (1:1000) as the secondary antibody. The number of brown granules in each high-powered field (magnification: ×400) was quantified as the number of positively stained cells or nuclei. The measurements were expressed as the percentage of positively stained cells or the ratio of nuclei to the total cells observed in 8-10 digital images per animal.

### Western blot

Protein extracts obtained from the lung tissues were obtained 12 h after LPS administration. Western blot analysis of the extracts was performed as previously described (19). In brief, the membranes were incubated for 1 h at room temperature in the presence of the primary antibody (dilution, 1:1000), washed three times with TTBS buffer, and incubated at room temperature for 1 h in the presence of the secondary antibody (dilution, 1:5000). After the blots were washed three times with TTBS buffer, they were developed and exposed by enhanced chemiluminescence.

### Plasma levels of cytokines (TNF-α, IL-6 and HMGB1)

Blood samples were collected via cardiac puncture at 3, 6, 9, and 12 h after the administration of LPS. All rats were euthanized with phenobarbital sodium before blood collection. The collected blood samples were centrifuged at 377.3 *g* for 10 min at 4°C, and the plasma supernatant retrieved was stored at -20°C until further analysis. The plasma levels of TNF-α and IL-6 were detected using solid-phase sandwich enzyme-linked immunosorbent assay (ELISA) kits specific for the detection of these factors, and the absorbance was measured at 450 nm by a plate reader (BioTek ELx800, USA).

### Statistical analysis

Data are reported as the means±SE or SD. Intergroup comparisons were made using one-way analysis of variance (ANOVA), followed by Student-Newman-Keuls q-test (SNK-q). The differences were considered to be significant if P<0.05.

## Results

### Effect of GAL on LPS-induced mortality

Ten rats in each group were investigated for survival analysis at 72 h after LPS injection. As expected, all the rats in the control group survived. However, in the LPS group, 50% of the rats died within 24 h while an additional 30% died within 72 h; thus, the total mortality rate in this group was 80% in contrast to the LPS+GAL group where the mortality rate was 10%.

### GAL pre-treatment attenuated LPS-induced pathological changes in lung tissue

**Figure 1:**
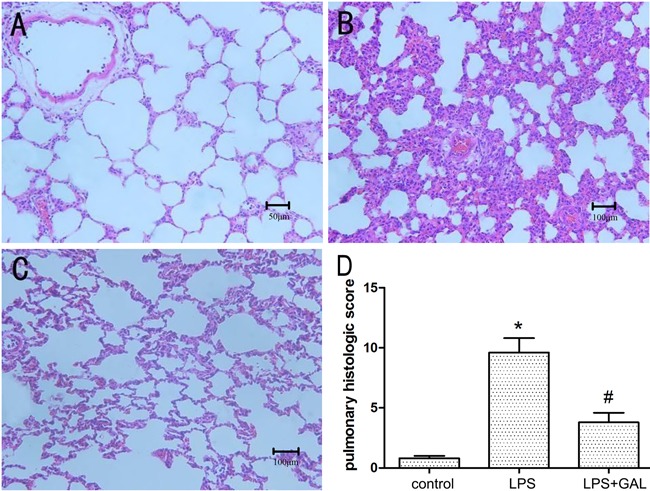
Histopathological changes in lung tissue samples of the three groups. Hematoxylin and eosin stain (×200 magnification). *A*, Control group (n=5): normal lung structure (bar 50 μm). *B*, LPS group (n=3): increased alveolar wall thickness, edema, bleeding, and infiltration of inflammatory cells (bar 100 μm). *C*, LPS+GAL group (n=5): mild structure destruction and inflammatory infiltration. (bar 100 μm). *D*, comparison of the pulmonary histological scores of the 3 groups. GAL: galantamine; LPS: lipopolysaccharide. ^*^P<0.05, LPS group compared to control group; ^#^P<0.05, LPS+GAL group compared to LPS group (ANOVA).

The control group showed no significant histological alterations. The rats exposed to LPS showed increased alveolar wall thickness, edema, bleeding, and infiltration of inflammatory cells at 12 h after LPS administration, indicating the occurrence of ALI. Rats pre-treated with GAL showed significantly less inflammation and distortion of pulmonary architecture after LPS administration as compared to those not treated with GAL. (Figure 1A-C; H&E staining, ×200 magnification). The total scores of the pulmonary histological changes in the groups indicated that the degree of pulmonary injury in the LPS+GAL group was significantly less than that in the LPS group (P<0.05, Figure 1D).

### Effect of GAL pre-treatment on W/D ratio

**Figure 2:**
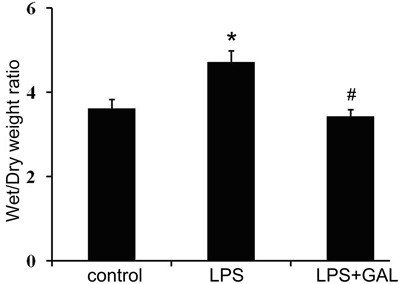
Comparison of the wet/dry weight ratio. The extent of pulmonary edema was assessed using the wet/dry ratio at 12 h after lipopolysaccharide (LPS) infusion. Control group: n=5; LPS group: n=3; LPS+galantamine (GAL) group: n=5. Data are reported as the means±SD. ^*^P<0.05, LPS group compared to control group; ^#^P<0.05, LPS+GAL group compared to LPS group (ANOVA).

The LPS group had a significantly higher W/D ratio than the control group, indicating the presence of pulmonary edema (P<0.05). However, the W/D ratio in the LPS+GAL group was significantly lower than that in the LPS group, indicating that GAL attenuated the degree of pulmonary edema induced by LPS (P<0.05, Figure 2).

### Effect of GAL on LPS-induced MPO activity

**Figure 3:**
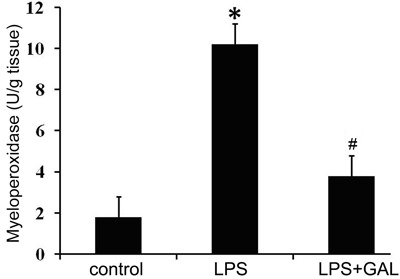
Effect of galantamine (GAL) on myeloperoxidase (MPO) activity in rat lungs. Neutrophil infiltration was assessed in terms of MPO activity level at 12 h after lipopolysaccharide (LPS) administration. Control group: n=5; LPS group: n=3; LPS+GAL group: n=5. Data are reported as the means±SD. ^*^P<0.05, LPS group compared to control group; ^#^P<0.05, LPS+GAL group compared to LPS group (ANOVA).

The level of MPO activity in the LPS group was significantly higher than that in the control group (10.2±1.12 *vs* 1.8±0.35 U/g tissue, P<0.01). However, the MPO activity level in the LPS+GAL group was significantly lower than that in the LPS group (3.8±0.62 U/g tissue, P<0.05 *vs* the LPS group), indicating that GAL inhibited MPO activity (Figure 3).

### GAL suppressed LPS-induced HMGB1 expression in lungs

**Figure 4:**
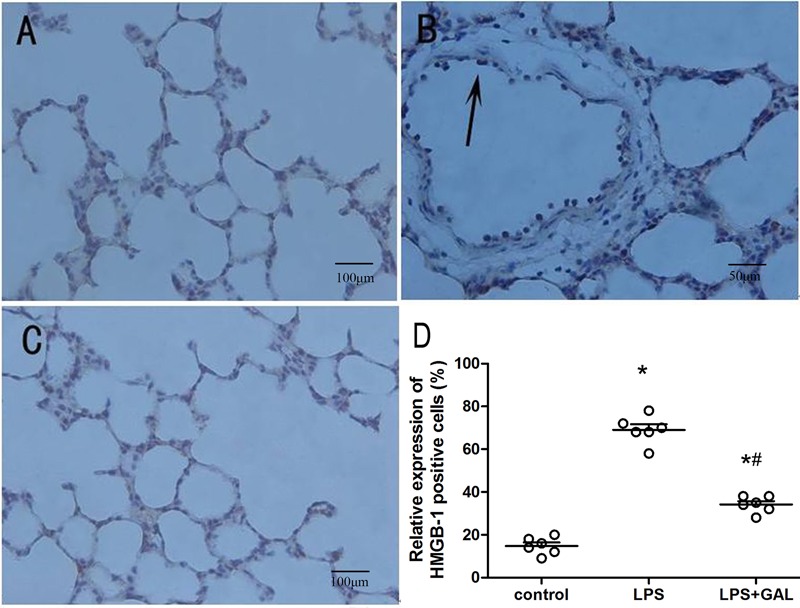
Immunohistochemical expression of high-mobility group box 1 (HMGB1) protein in rat lungs. *A*, Control group: n=5 (bar 100 μm). *B*, lipopolysaccharide (LPS) group: n=3 (bar 50 μm). *C*, LPS+galantamine (GAL) group: n=5 (bar 100 μm). The arrow indicates cells that stained positive for HMGB1 expression. Representative photomicrographs of lung immunohistochemical analysis (400×) show the increased redistribution of HMGB-1 expression from the nucleus to the cytoplasm and extracellular areas in bronchial epithelial cells, alveolar epithelial cells, and inflammatory cells. *D*, Scatter plot of HMGB-1-positive (+) cells (%) in lung tissues. ^*^P<0.05, LPS and LPS+GAL groups compared to control group; ^#^P<0.05, LPS+GAL group compared to LPS group (ANOVA test).

**Figure 5:**
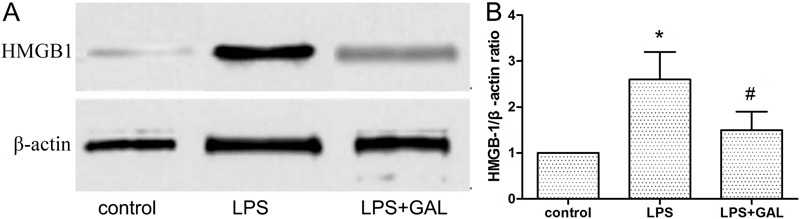
Western blot analysis of high-mobility group box 1 (HMGB1) levels in rat lung tissue at 12 h. *A*, The concentrations of HMGB1 and β-actin in lung tissue were determined by Western blot analysis at 12 h. Results of a representative experiment are shown. *B*, Galantamine (GAL) down-regulated the lipopolysaccharide (LPS)-induced elevation of HMGB1 expression. The results show the HMGB1/β-actin ratio obtained from the Western blots. Control group: n=5; LPS group: n=3; LPS+GAL group: n=5. Data are reported as the means±SE. ^*^P<0.05, LPS group compared to control group; ^#^P<0.05, LPS+GAL group compared to LPS group (ANOVA).

At 12 h, the expression levels of HMGB1 protein in the LPS group were considerably higher than those in the control group, while the expression levels in the LPS+GAL group were markedly lower than those in the LPS group (Figure 4). This indicated that GAL down-regulated the LPS-induced increase in HMGB1 expression, similar to that indicated by the Western blot analysis (Figure 5).

### GAL down-regulated the release of pro-inflammatory cytokines

**Figure 6:**
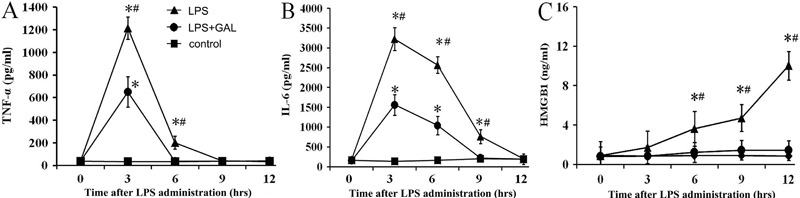
Changes in the levels of pro-inflammatory cytokines: *A*, tumor necrosis factor (TNF)-α, *B*, interleukin (IL)-6, and *C*, high-mobility group box 1 (HMGB1). Control group: n=5 for each time point; LPS group: n=5 (3 and 6 h), n=4 (9 h) and n=3 (12 h); LPS+GAL group: n=5 for each time point. GAL: galantamine; LPS: lipopolysaccharide. Data are reported as the means±SE. ^*^P<0.05, LPS and LPS+GAL groups compared to control group; ^#^P<0.05, LPS group compared to LPS+GAL group (ANOVA).

In the LPS group, the levels of TNF-α and IL-6 increased sharply after LPS administration and reached peak levels at 3 h. Thereafter, the levels decreased gradually to baseline levels at 12 h. However, the levels of the late stage pro-inflammatory cytokine HMGB1 increased gradually and reached a peak at 12 h. In contrast, the rats pretreated with GAL had significantly lower levels of TNF-α (LPS+GAL group *vs* LPS group: P<0.05 at 3 and 6 h), IL-6 (LPS+GAL group *vs* LPS group: P<0.05 at 3, 6 and 9 h), and HMGB1 (LPS+GAL group *vs* LPS group: P<0.05 at 6, 9 and 12 h) at the indicated time points (Figure 6).

## Discussion

In the present study, a rat model of ALI was successfully established by the intravenous administration of LPS. We found that LPS exposure caused a dramatic increase in the MPO activity level and W/D ratio, reflecting the occurrence of neutrophil infiltration and pulmonary edema. Furthermore, histopathological analysis revealed the loss of epithelial integrity. Taken together, these manifestations confirmed the development of LPS-induced ALI. Interestingly, pretreatment with GAL not only improved the survival of LPS-exposed rats, but also reduced the extent of histopathological changes, neutrophil infiltration, and secretion of proinflammatory cytokines in rat lung tissue.

Gram-negative sepsis is the most common risk factor of acute respiratory distress syndrome. LPS is the principal component of the outer membrane of gram-negative bacteria and is a potent stimulator of rapid pro-inflammatory cytokine production. The elevated expression of TNF-α, IL-1β, and IL-6 is an important step in the pathogenesis of ALI and acute respiratory distress syndrome (20). Moreover, in the case of humans with ALI or sepsis, a persistent elevation of these cytokines was associated with a poor prognosis (21). However, previous clinical studies have shown that anti-inflammatory agents such as monoclonal anti-TNF antibodies, IL-1 receptor antagonists, and TNF-receptor fusion proteins fail to prolong patient survival (22,23). This failure may be explained by the fact that the levels of TNF-α and IL-1β are elevated during the early stage of sepsis and recover at the late stage of disease. Consistently, our study showed that the levels of TNF-α and IL-6 reached a peak at 3 h after LPS administration and then returned to baseline levels thereafter. The persistence of lung injury suggests that other late stage downstream pro-inflammatory cytokines may be involved in the progression of ALI.

HMGB1 has been reported to play a crucial role in the inflammatory response and pathogenesis of LPS-induced lung injury (24). HMGB1 is a nuclear protein that functions as a DNA chaperone protein in normal cells and promotes interactions between proteins and DNA. In addition, HMGB1 is thought to be a late mediator of sepsis. In the current study, we observed a delayed elevation of HMGB1 in contrast to TNF-α and IL-6. This finding is consistent with those of a previous study, which showed that bile TNF-α concentrations peaked at 3 h after LPS challenge while HMGB1 concentrations showed a significant increase from 8 to 12 h (8). Furthermore, the intratracheal administration of HMGB1 induced neutrophil infiltration in lung tissues and increased the pulmonary expressions of pro-inflammatory cytokines such as TNF-α, IL-1β, and macrophage inflammatory protein-2 (7). However, in the present study, we did not observe an increase in TNF-α and IL-6 levels subsequent to the increase in HMGB1 expression. This may be due to several reasons. First, changes in the cytokine levels were monitored only for 12 h in this study. Therefore, it is not possible to comment whether fluctuations in the TNF-α and IL-6 levels occurred after this time point. Second, it is unclear whether the amount of circulating HMGB1 generated in this study was sufficient to enhance the expression of other inflammatory cytokines.

Our findings indicated that GAL pretreatment inhibited HMGB1 expression in the plasma and lung tissues. GAL may thus mitigate the inflammatory response and lung injury symptoms through its inhibitory effect on HMGB1 levels in rats. Recent studies have shown that extracellular HMGB1 participated in signaling via receptor for advanced glycated end products (RAGE) and/or members of the toll-like receptor (TLR) family, namely, TLR2 and TLR4. RAGE was identified as the major functional receptor involved in the pro-inflammatory effect of HMGB1 in rodent macrophages. Furthermore, the activation of RAGE and TLR triggers inflammatory responses mediated by nuclear factor (NF)-κB. Because TLR4 recognizes LPS from gram-negative bacilli, the interactions between HMGB1 and TLR4 may explain how HMGB1 triggers inflammatory responses similar to those elicited by LPS. In addition, studies have shown that anti-HMGB1 antibodies can prevent death in animal models of sepsis, hepatic ischemia-reperfusion injury, and rheumatoid arthritis (25,26). This beneficial effect can be explained because the administration of anti-HMGB1 antibodies before or after endotoxin treatment causes a significant decrease in the extent of endotoxin-induced neutrophil infiltration and lung edema (20). Thus, our findings confirmed the anti-inflammatory effect of GAL. However, further studies are necessary to identify the downstream mechanisms.

Because MPO is mainly expressed in the primary granules of neutrophils, elevated levels of MPO levels in tissues imply the presence of neutrophil infiltration within lung parenchyma or alveolar spaces (27). In this study, we observed that GAL pretreatment led to a significant suppression of MPO activity in lung tissues, suggesting that GAL inhibited the neutrophil infiltration into the lung parenchyma and alveolar spaces in the setting of LPS-induced ALI.

The most common adverse effects reported with the use of GAL are gastrointestinal disturbances, such as nausea, vomiting, diarrhea, anorexia, and abdominal pain. However, these effects have been reported for the oral administration of GAL and not when administered as a single *ip* injection, as used in this study. The current study demonstrated that GAL elicited the following changes in rats with ALI: 1) improved early-stage survival rate, 2) ameliorated histopathological changes that indicate lung injury, and 3) inhibited the release of pro-inflammatory cytokines. Taken together, these results suggest that GAL might be a potential candidate for the treatment of LPS-induced ALI.

This study had some limitations. First, the effect of GAL was assessed for a short duration only. Second, the protocol in this study involved pretreatment of the rats with GAL before the administration of LPS; this is not consistent with disease in clinical settings. Third, this study did not clarify whether the continuous administration of GAL enhanced its protective effect. Thus, long-term investigations designed in accordance with clinical settings are necessary to verify the potential of GAL for the management of sepsis-induced ALI.

In conclusion, the current study showed that GAL exerted a protective effect against LPS-induced ALI, which appeared to be mediated by inhibiting the release of proinflammatory cytokines, especially HMGB1.
